# Coordination of Ribosomal Protein and Ribosomal RNA Gene Expression in Response to TOR Signaling

**DOI:** 10.2174/138920209788185261

**Published:** 2009-05

**Authors:** LiJuan Xiao, Anne Grove

**Affiliations:** Department of Biological Sciences, Louisiana State University, Baton Rouge, LA 70803, USA

**Keywords:** Rapamycin, TORC1, HMO1, high mobility group, yeast, RP gene, rDNA.

## Abstract

Cells grow in response to nutrients or growth factors, whose presence is detected and communicated by elaborate signaling pathways. Protein kinases play crucial roles in processes such as cell cycle progression and gene expression, and misregulation of such pathways has been correlated with various diseased states. Signals intended to promote cell growth converge on ribosome biogenesis, as the ability to produce cellular proteins is intimately tied to cell growth. Part of the response to growth signals is therefore the coordinate expression of genes encoding ribosomal RNA (rRNA) and ribosomal proteins (RP). A key player in regulating cell growth is the Target of Rapamycin (TOR) kinase, one of the gatekeepers that prevent cell cycle progression from G1 to S under conditions of nutritional stress. TOR is structurally and functionally conserved in all eukaryotes. Under favorable growth conditions, TOR is active and cells maintain a robust rate of ribosome biogenesis, translation initiation and nutrient import. Under stress conditions, TOR signaling is suppressed, leading to cell cycle arrest, while the failure of TOR to respond appropriately to environmental or nutritional signals leads to uncontrolled cell growth. Emerging evidence from *Saccharomyces cerevisiae *indicates that High Mobility Group (HMGB) proteins, non-sequence-specific chromosomal proteins, participate in mediating responses to growth signals. As HMGB proteins are distinguished by their ability to alter DNA topology, they frequently function in the assembly of higher-order nucleoprotein complexes. We review here recent evidence, which suggests that HMGB proteins may function to coordinate TOR-dependent regulation of rRNA and RP gene expression.

## INTRODUCTION

Eukaryotic cell growth is regulated by intricate signaling pathways in response to nutrient levels, environmental stress and the presence of growth factors. Several protein kinases contribute to such signaling, including the target of rapamycin (TOR) kinase, a highly conserved serine/threonine kinase that plays a central role in the control of cell growth (reviewed in [1-3]). TOR is structurally and functionally conserved in all eukaryotes, and in mammalian cells, its dysregulation has been linked to many forms of cancer (reviewed in [4-7]). When growth conditions are favorable, TOR is active; this results in cells supporting ribosome biogenesis, translation initiation and nutrient import. Under stress conditions, TOR signaling is suppressed, resulting in cell cycle arrest. The failure of TOR to respond appropriately to environmental factors or nutritional signals leads to uncontrolled cell growth, as TOR signaling controls many aspects of cellular metabolism, including initiation of translation and transcription.

Up to 80% of the transcriptional machinery in *Saccharomyces cerevisiae* is dedicated to synthesis of ribosomal components - rRNA by RNA polymerase (Pol) I, ribosomal proteins by Pol II and 5S ribosomal RNA and tRNA by Pol III - while in the proliferating mammalian cell, as much as 50% of nuclear transcription is committed to this goal. What has also becomes increasingly evident is that ribosome biogenesis is an important determinant of cell growth; to maintain steady growth in response to favorable conditions, about 2000 ribosomes need to be synthesized per minute. While the role of TOR in regulating the synthesis of ribosomal components is well established, the mechanism by which all three polymerases are coordinately regulated in response to appropriate stimuli remains to be fully elucidated. As reviewed here, emerging evidence points to a role for high mobility group (HMGB) proteins in mediating such coordinated regulation in response to TOR signaling.

## TOR COMPLEXES

Both yeast and mammals contain two functionally and structurally distinct TOR complexes, TOR complex 1 (TORC1) and TOR complex 2 (TORC2), each composed of TOR as well as subunits that determine downstream substrates [8]. TORC1 controls cell growth *via* a rapamycin-sensitive signaling pathway [9-11] while TORC2 controls the organization of the actin cytoskeleton through a rapamycin-insensitive signaling pathway [12-15]. While higher eukaryotes encode a single TOR homolog, yeast encodes two TOR proteins, TOR1 and TOR2, either of which may become components of TORC1. TOR1 and TOR2 are therefore functionally redundant as components of TORC1, which responds to rapamycin by generating cellular responses akin to those induced by stress or starvation, including down-regulation of translation, inhibition of ribosome biogenesis, and specific changes in gene expression. In contrast, yeast TOR2 is uniquely required for mediating the cell cycle-dependent polarization of the actin cytoskeleton and is the kinase component of TORC2 [13, 16, 17].

The ~280 kDa TOR proteins share 40-60% sequence identity and contain several domains. The N-terminal domain contains a number of HEAT motifs (for Huntington, EF3, A subunit of PP2A, TOR1), required for proper subcellular localization [18]. A central FAT domain (for FRAP, an earlier name for mammalian TOR, ATM, TRAP) may mediate protein-protein interactions [18, 19]. The C-terminal domain harbors the serine/threonine protein kinase activity. TOR proteins belong to the phosphatidyl inositol kinase-related kinase (PIKK) family, as their catalytic domain resembles that of the phosphatidyl inositol 3-kinases (PI3K). The kinase domain is immediately preceded by the FKBP12-rapamycin binding domain (FRB). Both TORC1 and TORC2 are multimeric complexes (Fig. (**1**); [14, 20]).

The response to rapamycin requires that the drug first combine with the intracellular receptor FKBP12 [9, 21]. FKBP12-rapamycin binds TORC1 to inhibit its function. Indeed, TOR was originally identified on the basis of mutations that confer resistance to rapamycin, which is a macrocyclic lactone produced by *Streptomyces hygroscopicus* as an antifungal agent. Treatment of yeast cells with rapamycin phenocopies nitrogen starvation or inactivation of TOR by leading to down-regulation of protein synthesis and upregulation of macroautophagy (delivery of cytoplasmic contents to lysosomes and vacuoles). The mechanism by which FKBP12-rapamycin inhibits TORC1 is not known but has been proposed to include blockage of substrate access or dissociation of TOR from associated proteins [22, 23]. Proteins associated with TOR in yeast TORC1 have been identified as KOG1, TCO89, and LST8, of which only TCO89 has no obvious mammalian homolog (Fig. (**1**)); KOG1 deficiency in yeast resembles the phenotype of rapamycin treatment, suggesting that KOG1 is a positive regulator of TOR, an inference also reached with regard to LST8 [10, 24]. Knockdown of the mammalian KOG1 homolog (Raptor), which interacts with downstream targets of TOR, likewise emulates treatment with rapamycin [22, 25, 26].

## TARGETS OF TORC1

Several upstream signaling cues, including growth factors in mammals, nutrients, and environmental stress such as hypoxia and DNA damage regulate TORC1 signaling. Major targets of TORC1 include components of the translation machinery (reviewed in [3]). Such downstream targets include the general translation initiation factor 4E-BP which functions to regulate availability of the initiation factor eIF4E, essential for cap-dependent initiation of translation [27-29]. Another well-established target of the TORC1 complex is the S6 protein kinase S6K1, which phosphorylates ribosomal protein S6, an event that controls elongation factor 2 kinase, but probably not translation of mRNAs with a 5'-oligopyrimidine tract as originally suggested [30-32].

### TORC1-Mediated Regulation of rDNA Transcription

In addition to numerous roles in regulation of translation, TORC1 regulates transcription of all genes whose products are involved in ribosome biogenesis. Production of rRNA accounts for at least 60% of total yeast transcriptional activity during normal growth, so it is a highly energy- and nutrient-consuming process. To conserve resources, cells must therefore limit the production of new ribosomes during nutrient starvation. Accordingly, both synthesis and the subsequent processing of 35S precursor rRNA is regulated by the TOR signaling pathway in both mammalian and yeast cells [33, 34].

rDNA transcription is confined to the nucleolus, where rDNA genes are arranged as hundreds of head-to-tail repeats. The primary rRNA transcript is then processed into the mature rRNAs in the nucleolus, which is also the site of ribosome biogenesis. A recent study showed that rapamycin and nutrient starvation causes rapid delocalization of RNA Pol I from the nucleolus, suggesting a possible mechanism for the regulation of rDNA transcription by TOR [35]. These authors also found that rDNA chromatin undergoes dramatic remodeling and becomes condensed in the presence of rapamycin. Notably, the rapamycin-mediated inhibition of rDNA transcription was blocked by a mutation in RPD3, a histone deacetylase subunit of the RPD3-SIN3 complex. Since RPD3–SIN3 histone deacetylase is important for regulation of chromatin condensation in yeast, and since both RPD3 and SIN3 were found to be rapamycin sensitive in a genomic rapamycin-sensitivity screen, these findings suggest that RPD3-mediated histone H4 deacetylation at rDNA chromatin may be a critical step in rDNA transcriptional regulation [36, 37]. Further supporting a role for chromatin remodeling in TOR-mediated rDNA gene activity is that RSC9, a component of the chromatin remodeling complex RSC (Remodels the Structure of Chromatin), is differentially localized to genomic DNA on TOR inactivation [38]. That RPD3 was also reported not to be required for rapamycin-mediated inhibition of Pol I points to mechanisms beyond chromatin remodeling [39].

Like Pol II and Pol III, Pol I requires auxiliary factors to mediate promoter recognition; for mammalian Pol I, preinitiation complex formation requires the upstream binding factor UBF and the promoter selectivity factor TIF-IB/SL1 (TIF-IB in mouse; SL1 in humans) [40-42]. Yeast does not encode a UBF homolog, instead the HMGB protein HMO1 has been reported to be a component of the Pol I machinery [43], and yeast Pol I transcription also requires the multifunctional complex UAF that binds upstream of the core promoter [44, 45]. UBF contains several HMG boxes, which result in DNA wrapping about the UBF dimer to bring the core promoter and upstream control element UCE into proximity, creating an “enhancesome” [46, 47]. It is thought that this DNA conformation is required for productive interactions between UBF and TIF-IB/SL1 that manifest in recruitment of Pol I by UBF and stabilization of TIF-IB/SL1 [48]. UBF contacts Pol I directly through interaction with PAF53, the mammalian homolog of yeast RPA49, and TIF-IB/SL1 interacts with TIF-IA, the mammalian homolog of yeast Rrn3 [49]. TIF-1A/Rrn3 is a regulatory factor that associates with initiation-competent subpopulations of Pol I [50, 51].

In both yeast and mammals, TOR controls transcription by RNA polymerase I *via* the transcription factor TIF-IA/Rrn3, as inactivation of TOR impairs formation of the transcription initiation complex [34, 51-53]. Using chromatin immunoprecipitation assays, it was found that rapamycin treatment causes a decrease in the level of Rrn3-Pol I complex at the yeast rDNA promoter and coding region, similar to what occurs in stationary phase, suggesting that the decrease in transcriptional activity of individual active genes in stationary phase is mediated by the TOR signaling system through Rrn3-dependent polymerase recruitment [52]. Thus, the Pol I-Rrn3 interaction may be a major target of TOR-dependent regulation of rDNA transcription [51].

Changes in UBF phosphorylation are key to modulating rDNA activity during cell cycle progression; for example, phosphorylation of the C-terminal activation domain of UBF by casein kinase II (CKII) facilitates the interaction between UBF and TIF-IB/SL1 [54]. Stimulation of rDNA transcription by mTOR requires both activation of S6K1 and phosphorylation of the UBF C-terminal domain (but not directly by S6K1; [56]). By contrast, rapamycin treatment leads to the rapid dephosphorylation of the UBF C-terminal domain, which significantly reduces its ability to interact with SL1 [55].

In addition to signaling functions, including the control of cellular localization of specific transcription factors as discussed below, TOR1 itself is dynamically distributed in the cytoplasm and nucleus in yeast. TOR1 nuclear localization is nutrient-dependent and rapamycin sensitive, and starvation or treatment with rapamycin causes TOR1 to exit from the nucleus. TOR1 nuclear localization is critical for 35S rRNA synthesis, but not for the expression of ribosomal protein genes [56]. Thus, effects of TOR signaling on rRNA gene activity appear to be exerted at the levels of both chromatin remodeling and transcription factor activity, perhaps governed by the cellular localization of TOR.

### TORC1-Mediated Regulation of RP Gene Expression

Ribosomal protein (RP) genes in eukaryotes are also regulated in response to growth stimuli and environmental stress. Global transcription profiling revealed that RP gene transcription is drastically repressed in response to rapamycin [33, 57]. As for rRNA gene activity, chromatin remodeling has also been implicated in regulation of RP gene expression in response to TOR signaling. For example, Rohde and Cardenas found that expression of RP genes coincides with recruitment of the ESA1 histone acetyl transferase to RP gene promoters, while inhibition of TOR with rapamycin releases ESA1 from RP gene promoters and leads to histone H4 deacetylation. Genetic and biochemical evidence demonstrated that the chromatin deacetylation complex RPD3-SIN3, also implicated in regulating rDNA gene activity, contributes to the repression of RP gene expression in response to TOR inhibition by rapamycin or nutrient limitation [58, 59].

A number of transcription factors have been implicated in regulation of RP gene expression. Genome-wide detection of transcription factor binding revealed that the Forkhead-like transcription factor FHL1, originally isolated as a multicopy suppressor of RNA Pol III, is found at yeast RP gene promoters [60, 61]. Subsequent studies found that FHL1 may function either as a repressor or activator of RP gene transcription, depending on whether it associates with the corepressor CRF1 (Co-repressor with FHL1) or the coactivator IFH1 (Interacts with Forkhead). This decision is made based on the subcellular localization of CRF1, which is in turn determined by its phosphorylation state. TOR controls the subcellular localization of protein kinase A (PKA) and the PKA-regulated kinase YAK1 for which CRF1 is a substrate [62-64]; in actively growing cells, TORC1 negatively regulates the activity of YAK1, keeping CRF1 in the cytoplasm, while inhibition of TORC1 results in activation of YAK1, which in turn phosphorylates CRF1. Phosphorylated CRF1 translocates to the nucleus where it competes with IFH1 for binding to FHL1, which is constitutively bound at RP gene promoters [63]. The essential protein IFH1 is recruited to RP gene promoters through the forkhead-associated domain of FHL1 [62, 64, 65]; FHL1 and IFH1 were shown to associate almost exclusively with RP promoters, and the level of IFH1 associated with RP promoters suggested to determine the level of transcription [64]. Accordingly, IFH1 is thought to be an essential regulator of RP gene transcription (Fig. (**2**)). That additional factors must contribute to TOR-mediated regulation of RP gene activity was indicated by the observation that CRF1 may not be essential for rapamycin-dependent down-regulation of RP gene activity, depending on strain background, and that simply tethering of FHL1 to RP gene promoters is insufficient to cause transcriptional activation, in spite of the fact that such tethered FHL1 does recruit IFH1 [66].

Although yeast FHL1 has a domain that is related to the DNA binding domain of the Drosophila Forkhead protein, FHL1 was reported not to bind DNA *in vitro* [67]. Thus, the binding of FHL1 to an RP promoter is likely to be either indirect or facilitated by other proteins. One such candidate for FHL1 recruitment is the repressor-activator protein 1 (Rap1), long known to be involved in the transcription of RP genes; most RP genes contain Rap1 binding sites at their promoters [68, 69]. However, Rap1 binds to many sites in the yeast genome and acts as an activator at some and as a repressor at others, as its name implies. Further, not all RP gene promoters contain Rap1 binding sites [70], suggesting that additional protein(s) contribute to FHL1 recruitment.

The complexity of the TOR pathway is further underscored by the reported involvement of yet another transcription factor, Sfp1, a key cell size regulator, in regulating RP gene expression in response to nutrients and stress [71]. Under optimal growth conditions, Sfp1 is localized to the nucleus, bound to the promoters of RP genes where it activates RP gene expression. In response to inhibition of TOR signaling, Sfp1 is released from RP gene promoters, and down-regulation of RP gene transcription occurs concomitant with Sfp1 leaving the nucleus. That Sfp1 remains localized to the nucleus in a strain constitutively expressing high PKA activity in spite of rapamycin treatment suggests that Sfp1 may be regulated by the branch of the TOR pathway that depends on PKA, similar to the regulation of CRF1 localization [63, 71]. Analysis of genetic interactions further suggested that Sfp1 influences RP gene transcription *via *FHL1 and IFH1, and that the association of IFH1 and FHL1 with RP gene promoters was reduced by approximately four-fold and two-fold, respectively, in *sfp1Δ* cells [72]. As for the role of CRF1 in communicating downstream signals of the TOR pathway, the role of Sfp1 may be strain-dependent, as a subsequent genome-wide ChIP analyses suggested only limited association of Sfp1 with RP genes [73]. Thus, while different transcription factors have been variously implicated in mediating responses to TOR signaling, suggesting the existence of more than one mechanism by which such signals are converted to changes in gene activity, signaling through FHL1 appears to be the primary pathway.

### Coordination of rRNA and RP Gene Expression in Response to TOR Signaling

As summarized above, an effect of TOR inactivation on rRNA and RP gene expression has been well established, although the molecular basis for the response remains to be fully elucidated. Notably, recent evidence has finally furnished a link that may provide a clue to the mechanism by which transcriptional activity of the different RNA polymerase machineries may be coordinated in response to TOR signaling. This link comes in the form of the yeast HMGB homolog HMO1. HMGB proteins are conserved architectural proteins that bind DNA without sequence specificity, but with preference for DNA with structural distortions. As for mammalian HMGB homologs, HMO1 is composed of two HMG boxes, box A and box B. Box A has low affinity for DNA but is required for DNA structural specificity and for DNA bending, whereas box B is required for high affinity DNA binding [74, 75]. Genetic analysis showed that HMO1 is important for maximal transcription of rDNA [43]. While the molecular basis for the involvement of HMO1 in Pol I transcription is unknown, it is noteworthy that HMO1 overexpression suppresses an *rpa49Δ* mutation, as RPA49 corresponds to the mammalian PAF53 which interacts directly with the HMG-box-containing transcription factor UBF; as noted above, yeast does not encode a UBF homolog [43, 48, 49]. It is therefore tempting to speculate that HMO1 may serve to configure the yeast Pol I promoter for optimal transcriptional activity, akin to the enhancesome structure proposed for UBF [46, 47].

A seminal study by Hall and coworkers demonstrated that HMO1 associates throughout the rDNA locus [76]. Their study also confirmed that HMO1 is required for maximal transcription of rRNA genes and demonstrated a role for HMO1 in rRNA maturation. Considering the localization of HMO1 throughout the rDNA locus, its reported role in rRNA processing, and recent evidence linking RNA Pol I transcriptional elongation with rRNA maturation, it is conceivable that HMO1 participates in coordinating these activities [77]. Importantly, HMO1 was also found to associate with most RP gene promoters and to do so in a Rap1-dependent fashion [76]. While Rap1 appears to be required for association of HMO1 with RP gene promoters, it is not sufficient, as evidenced by its localization to other genomic loci to which HMO1 does not associate. Notably, HMO1 association is reduced in an *fhl1Δ* strain, while inactivation of *HMO1* completely eliminates association of FHL1 and IFH1 with RP genes [76]. These data indicate that HMO1 and FHL1 bind cooperatively to RP gene promoters, and that Rap1 may promote this association.

Using a global genetic approach, Berger *et al.* subsequently reported that HMO1 specifically interacts with the Pol I-transcribed region of the rDNA locus, pointing to a role in elongation, and confirmed its association with a subset of RP gene promoters [78]. This is consistent with an independent report indicating that HMO1 associates with the 35S rRNA gene in an RNA Pol I-dependent manner [73]. Notably, HMO1 was seen to dissociate from both rDNA and some RP gene promoters upon rapamycin treatment, and HMO1 deletion was shown to reduce the TORC1-dependent repression of RP gene expression [78]. These results clearly link HMO1 to the rapamycin-dependent TOR pathway and suggest that HMO1 is involved in coordinating rDNA transcription by Pol I and RP gene expression by Pol II in response to TOR signaling.

## CONCLUSION

The molecular mechanisms underlying the role of HMO1 in coordinating rRNA and RP gene transcription in response to TOR-mediated signals remain to be determined. In the case of rDNA, HMO1 has been proposed to serve roles akin to those fulfilled by UBF in mammalian transcriptional initiation as well as in linking transcriptional elongation with pre-rRNA processing. Regardless of its specific function(s), it is clear that HMO1 abandons the rDNA locus when TORC1 is inhibited [78]. For UBF, dephosphorylation in response to rapamycin treatment is accompanied by reduced recruitment of Pol I [55]. Whether HMO1 is likewise subject to covalent modification in a rapamycin-dependent fashion remains to be determined.

At RP gene promoters, several transcription factors have been implicated in mediating downstream effects of TOR signaling. While the role of Sfp1 appears particularly uncertain, a consensus is emerging based on consistent reports from several groups (Fig. (**2**)). At most RP gene promoters, HMO1 appears to associate in a Rap1-dependent fashion, followed or accompanied by FHL1; however, at the promoters to which HMO1 only associates modestly, FHL1 binding appears independent of HMO1 [73]. The transcription factor IFH1 is essential, consistent with its reported role as a co-activator of RP gene transcription; if IFH1 is absent, the co-repressor CRF1 would also encounter no competition for binding to FHL1, thus potentially abolishing even basal levels of RP transcription. Depending on genetic background, a rapamycin-mediated down-regulation of RP gene activity may still be seen when CRF1 is inactivated; however, the basis for this observation remains unknown and has been suggested to involve more than one gene product [66]. One possible clue derives from the observation that IFH1 is found to a significant extent in a complex named CURI, composed of casein kinase II (CKII), Utp22, Rrp7 and IFH1), a complex with which FHL1 also loosely associates [79]. Notably, both Utp22 and Rrp7 have been implicated in pre-rRNA processing, a process in which HMO1 is also involved. It is possible that TOR-dependent sequestration of IFH1 in the CURI complex limits its availability as an RP gene activator, causing down-regulation of RP gene expression on inhibition of the TOR pathway. Activation of rDNA transcription may lead to disruption of CURI as Utp22 and Rrp7 engage in pre-rRNA processing, thus releasing IFH1 to activate RP gene expression [79]. Whether HMO1 participates in this proposed scenario has not been addressed; however, HMO1 has been reported to interact directly with Utp22 [80], consistent with both proteins participating in pre-rRNA processing, suggesting the possibility that CURI may be recruited to both rRNA and RP genes by interaction with HMO1.

As HMO1 is non-sequence-specific in its DNA binding [74, 75], it is likely recruited to RP gene promoters by association with sequence-specific factors; that FHL1 fails to bind DNA i*n vitro*, even in the presence of Rap1 [67], combined with the co-dependence of HMO1 and FHL1 for binding to RP genes [76], suggests that HMO1 (or another factor, perhaps Sfp1) may be required to stabilize it on the DNA. HMO1 was reported either to have no effect or to cause a mild activation of RP gene transcription [76, 78]; such effects may be due to its interaction with TFIID, which has been reported to affect occupancy of TFIID at certain RP gene promoters as well as to participate in start site selection of a subset of Pol II genes [81]. As for rDNA, the mechanism by which HMO1 leaves the RP gene promoters following inhibition of the TOR pathway is not known.

## Figures and Tables

**Fig. (1):**
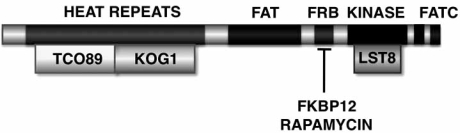
**Domain organization of TOR kinases.** Functional domains depicted include the N-terminal HEAT repeats, the central FAT domain, the kinase domain, and the C-terminal FRB domain to which FKBP12-rapamycin associates. Proteins associating with TORC1 include TCO89, KOG1, and LST1, of which only TCO89 has no apparent mammalian homolog.

**Fig. (2):**
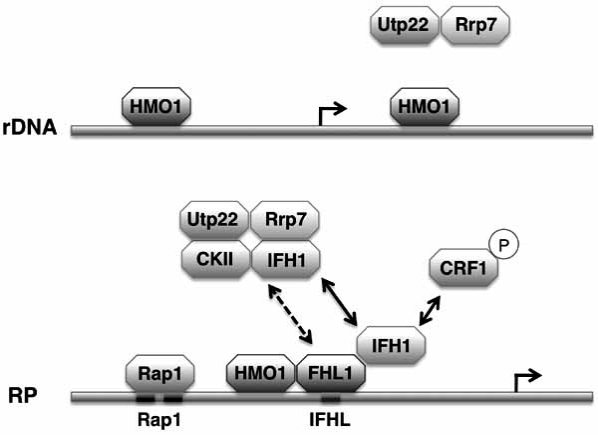
**HMO1 associates with both rRNA and RP gene promoters to coordinate gene expression in response to TORC1 activity.** On rDNA, HMO1 associates with both promoter and coding regions of transcriptionally active rDNA. Proposed functions include a role akin to that reported for mammalian UBP in transcriptional initiation and a role in coordinating transcriptional elongation with pre-rRNA processing, to which Utp22 and Rrp7 contribute. On most RP gene promoters, Rap1 sites are further upstream than on other Rap1-dependent genes, and association of Rap1 appears to be required for the subsequent cooperative binding of HMO1 and FHL1 at IFHL sites. When TORC1 is active, the coactivator IFH1 binds FHL1 to activate RP gene transcription. When TORC1 is inactive, phosphorylated CRF1 translocates to the nucleus to compete with IFH1 for binding to FHL1 and repress transcription. IFH1 is found in the “CURI” complex also containing Utp22, Rrp7, and CKII, with which FHL1 also associates. HMO1 dissociates from both classes of genes on inactivation of TORC1.

## ABBREVIATIONS

TOR: = Target of Rapamycin
TORC1: = TOR Complex 1
RP: = Ribosomal protein
HMG: = High mobility group
FHL1: = Forkhead like
IFH1: = Interacts with Forkhead
CRF1: = Corepressor with Forkhead
Pol: = Polymerase
UBF: = Upstream Binding Factor
